# Characteristics of myogenic response and ankle torque recovery after lengthening contraction-induced rat gastrocnemius injury

**DOI:** 10.1186/1471-2474-13-211

**Published:** 2012-10-30

**Authors:** Hongsun Song, Eisuke Ochi, Kihyuk Lee, Kenji Hiranuma, Koichi Nakazato

**Affiliations:** 1 Korea Institute of Sport Science, San223-19, Gongneung-2Dong, Nowon-gu, Seoul, 139-242, South Korea; 2 Laboratory of Health and Sports Sciences, Center for Liberal Arts, Meiji Gakuin University, 1518 Kamikurata-cho, Totsuka-ku, Yokohama, Kanagawa, 244-8539, Japan; 3 Graduate School of Health and Sport Science, Nippon Sport Science University, 7-1-1, Fukasawa, Setagaya-ku, Tokyo, 158-8508, Japan

**Keywords:** Muscle injury, Eccentric contraction, Regeneration, Myogenic factor

## Abstract

**Background:**

Although muscle dysfunction caused by unfamiliar lengthening contraction is one of most important issues in sports medicine, there is little known about the molecular events on regeneration process. The purpose of this study was to investigate the temporal and spatial expression patterns of myogenin, myoD, pax7, and myostatin after acute lengthening contraction (LC)-induced injury in the rat hindlimb.

**Methods:**

We employed our originally developed device with LC in rat gastrocnemius muscle (n = 24). Male Wistar rats were anesthetized with isoflurane (aspiration rate, 450 ml/min, concentration, 2.0%). The triceps surae muscle of the right hindlimb was then electrically stimulated with forced isokinetic dorsi-flexion (180°/sec and from 0 to 45°). Tissue contents of myoD, myogenin, pax7, myostatin were measured by western blotting and localizations of myoD and pax7 was measured by immunohistochemistry. After measuring isometric tetanic torque, a single bout of LC was performed *in vivo*.

**Results:**

The torque was significantly decreased on days 2 and 5 as compared to the pre-treatment value, and recovered by day 7. The content of myoD and pax7 showed significant increases on day 2. Myogenin showed an increase from day 2 to 5. Myostatin on days 5 and 7 were significantly increased. Immunohistochemical analysis showed that myoD-positive/pax7-positive cells increased on day 2, suggesting that activated satellite cells play a role in the destruction and the early recovery phases.

**Conclusion:**

We, thus, conclude that myogenic events associate with torque recovery after LC-induced injury.

## Background

Excessive lengthening contraction (LC) causes different types of damage to skeletal muscles including temporary muscle dysfunction, delayed onset muscle soreness (DOMS), and most importantly, muscle strain injury [1-4]. In particular, LC is reported to play a critical role in inducing muscle strain injury [3,4]. Although there have been many clinical and laboratory studies evaluating biomechanical mechanisms of muscle injury [3,5-8], the relationships between molecular events resulting from LC and functional recovery have not been fully understood.

In general, the healing process of muscle injury consists of 3 phases: the destruction phase, the repair phase, and the remodeling phase [9]. During these 3 phases, activation of the quiescent satellite cells (SCs) plays a key role [10,11]. Quiescent SCs expressing pax7 locate between the sarcolemma and the basal lamina in adult muscles [12,13]. When activated by the damage, SCs upregulate one of the myogenic regulatory factors (MRFs), myoD, and become activated SCs [14]. Activated SCs eventually become myoblasts, characterized by upregulated myogenin and downregulated pax7 [15-17]. These activated myogenic cells contribute to hyperplasia or hypertrophy of the injured muscles.

In the remodeling phase, myogenic cells, along with the reformation of precise connective tissues, assume a crucial role. The reorganization and construction of unstructured connective tissue can occur, leading to scar tissue formation and subsequent incomplete skeletal muscle regeneration [9]. Transforming growth factor-β1 (TGF-β1) is the primary factor contributing to fibrosis in various tissues [18], including skeletal muscles [19,20]. TGF-β and its superfamily, including myostatin [21,22], inhibit myogenic proliferation and differentiation via smad-mediated signal transduction [23]. Thus, the TGF-β superfamily plays a key role in the maturation of myogenic cells as well as precise connective tissue formation.

In myotoxic and contusion injury models, the recovery processes of muscle injuries have been examined. Beitzel et al. [24] showed that bupivacaine treated rat extensor digitorum longus (EDL) muscles had decreased EDL specific tension concomitant with decreased and regenerated muscle fiber cross-sectional area in hematoxylin-eosin (H & E) staining. Criso et al. [25] and Iwata et al. [26] demonstrated that contusion injury in the gastrocnemius resulted in significantly lower muscle strength. Crisco et al. showed that the tetanic tension of gastrocnemius returned to normal levels and, at the same time, there was no histological abnormality in H & E staining on 24 days after injury [25,26]. Iwata et al. also showed that locomotion and the isometric plantarflexion force returned to the normal levels 21 days after contusion injury in rat gastrocnemii, but they found that the muscle fiber size did not return to the normal value in 21 days [25,26]. There are still controversies between functional recovery and pathological findings in muscle injury. Especially, the relationships between myogenic proteins expressions and muscular functional recovery have not been fully examined. Further, such examinations have not been addressed to temporary muscle dysfunction caused by lengthening contraction, which frequently occur in sports situation.

With regard to strain injuries, a single LC or a passive stretch on an exposed muscle was used to elucidate the mechanisms of muscle strain injuries [3,6,7]. Since the exposed-muscle model includes highly invasive treatments (i.e. deep incision), it is hard to follow long term the physiological recovery process. In order to overcome these demerits, Best et al. [5] and Song et al. [8] showed that a minimally invasive method (without an incision) successfully induced muscle dysfunction and apparent pathologic damage in the tibia anterior or the gastrocnemius. Since their models avoided invasive treatment as much as possible, long-time the recovery processes can be examined, such as those in toxic and contusion muscle injury models.

This study investigated the relationship between the expression of myogenic proteins (myogenin, myoD, and pax7) and functional changes in the skeletal muscle after LC-induced injury. We hypothesized that the expressions of these proteins correlate with muscular torque recovery. To test our hypothesis, we employed the minimally invasive model of muscle injury reported by Song et al. [8]. Furthermore, we also examined myostatin expression in order to evaluate the processes of myogenic and connective tissue formation during the remodeling phase.

## Methods

### Animals

24 Wistar rats (age, 12 weeks; body mass, 260–280 g) were used in this study (CLEA Japan, Tokyo, Japan). Animals were housed in individually ventilated cage (IVC) systems (Tecniplast, Italy) maintained at 22–24°C with a 12-h light/dark cycle. Water and food were given *ad libitum* during the experiments. All procedures were approved by the Ethical Committee of the Nippon Sports Science University on the Use of Animal Subjects in Research (ID: 008-A01).

### Measurement of isometric tetanic torque

Isometric tetanic torque was measured as previously reported [8,20,27]. Maximal isometric plantar flexion torque was measured with a dynamometer at the ankle joint angle of 0° (defined as the angle at which the sole of the foot and the tibial bone are orthogonally positioned). Before the measurement, the right hindlimb was shaved for electrical stimulation. The rats were anesthetized and placed prostrate on a platform with their knee extended. The triceps surae muscle was stimulated supramaximally (pulse duration, 0.4 ms; frequency, 100 Hz; intensity, ~35 V) with self-adhesive surface electrodes connected to an electric stimulator and an isolator (SS-104 J; Nihon Koden, Japan). Measurements were made before the LC, and on days 2, 5, and 7 after the LC.

### Experimental method for muscle contraction-induced injury

To observe the regenerating and the recovery process, a single lengthening contraction-induced injury was induced *in vivo* to the rat gastrocnemius as described previously [8]. The right gastrocnemius was used for experimental intervention. The right hindlimb of all the animals were shaved, and each rat was then anesthetized with isoflurane (aspiration rate, 450 mL/min; concentration, 2.0%). At first, in the prone animal, the hindlimb was positioned by extending the knee and dorsiflexing the foot to 0°. The ankle angle of 0° was defined when the foot was aligned straight with the tibia. The limb and the body were secured with elastic bands to a steel support platform. The medial gastrocnemius was stimulated supramaximally by employing percutaneous electrical stimulation with self-adhesive surface electrodes connected to an electric stimulator and an isolator (SS-104 J; Nihon Koden, Japan). After achieving a fused tetanic activation, the foot was dorsiflexed from 0 to 45° at a velocity of 180°/sec, and then returned to the starting position. The stretching movement during the activation induced a single injurious LC to the medial gastrocnemius muscle. The animals were sacrificed prior to, and on days 2, 5, and 7 after the muscle injury (n = 6 at each time point) using isoflurane inhalation, and weighed their hindlimb triceps muscles (medial gastrocnemius, lateral gastrocnemius, plantaris, and soleus) were dissected and weighed.

For pathological evaluation, we excised a 2-mm thickness specimen in a transverse plane from gastrocnemius muscle bellies. The obtained specimen was embedded into OCT (Optimal Cutting Temperature) compound and immediately frozen in liquid N_2_. Other portions of medial gastrocnemius muscle (proximal and distal portions) were immediately frozen in liquid N_2_ for Western blot analysis. Specimens were stored at −80°C until analysis.

### Western blot analysis

Rapidly frozen medial gastrocnemius muscles were macerated under liquid N_2_, and homogenized in RIPA buffer (50 mM Tris-Cl (pH 7.4), 150 mM NaCl, 5 mM ethylenediaminetetraacetic acid (EDTA), 0.5% sodium dodecyl sulfate (SDS), 1% deoxycholate, 0.1% Triton X-100, 1% Nonidet P-40 (NP-40), 0.05% mercaptoethanol, 10 mg/mL phenylmethylsulfonyl fluoride (PMSF), 0.5 mg/mL leupeptin, 0.2 mg/mL aprotinin, and 1 mM Na_3_VO_4_). Protein concentrations were determined using Bio-Rad Protein Assay (Protein Assay II; Bio-Rad, Richmond, VA). The equivalent of 30 μg of total protein extract from each sample was mixed with sample buffer, boiled, and loaded onto a SDS-polyacrylamide gel (10%), and electrophoresed at 20 mA. The samples were electrophoretically separated at 180 mA for 90 min and then transferred onto polyvinylidene difluoride (PVDF) membranes (ATTO, Japan). The membranes were blocked for 1 h with phosphate-buffered saline (PBS) containing 5% skimmed milk followed by an overnight incubation at 4°C with a 1:1000 dilution of the following primary antibodies. All primary antibodies were incubated overnight at 4°C (dilution, 1:1000): monoclonal anti-myoD (DAKO, Capinteria, CA), monoclonal anti-myogenin (DAKO, Capinteria, CA), polyclonal anti-myostatin (Millipore, Billerica, MA), polyclonal anti-pax7 (Abcam, Cambridge, MA), and alpha-tubulin loading control (Abcam, Cambridge, MA). Membranes were washed 3 times for 10 min each at room temperature, followed by incubation with secondary horseradish peroxidase-conjugated goat anti-rat immunoglobulin G (IgG) or anti-rabbit IgG at a dilution of 1:10,000. The proteins were detected using chemiluminescence reagents (SuperSignal West Dura; Pierce Protein Research Products, Rockford, IL) with a chemiluminescence detector (AE6961; ATTO, Japan), and quantified using a personal computer with image analysis software (CS Analyzer; ATTO, Japan). The band densities were expressed relative to those obtained for the loading control.

### Immunostaining

Frozen medial gastrocnemius blocks were transversely sectioned with a Cryostat (CM 1500, Leica, Germany) at −20°C. Sections (10 μm) from the mid-belly portion of the medial gastrocnemius muscle were air dried for 15 min, then fixed using 4% paraformaldehyde in a 0.1 M phosphate buffer (pH 7.4) for 15 min. The sections were then washed in 0.1 M PBS and incubated with 0.1 M PBS containing 10% normal serum and 0.3% Triton X-100 for 1 h at RT to block nonspecific binding. For triple-immunofluorescence staining, sections were incubated simultaneously with primary antibodies diluted with 0.1 M PBS containing 5% normal serum, and 0.3% Triton X-100 for 20 h at 4°C. The primary antibodies used were polyclonal anti-myoD (1:200; DAKO, CA), polyclonal anti-myogenin (1:200; DAKO, CA), polyclonal anti-pax7 (Abcam, Cambridge, MA), polyclonal anti-laminin (a marker molecule for the basement membrane, 1:3000; Sigma-Aldrich, MO). The sections were washed in 0.1 M PBS and incubated with the secondary antibodies diluted in 0.1 M PBS containing 5% normal serum, and 0.1% Triton X-100 overnight at 4°C. Fluorescein-conjugated donkey anti-mouse IgG was used against mouse monoclonal primary antibodies and rhodamine-conjugated donkey anti-rabbit IgG was used against rabbit polyclonal antibodies (Chemicon, CA). To distinguish whether myoD-positive cells, triple immunostaining was performed co-localized with DAPI nuclei and pax7 to find out the ratio of myonuclei and satellite cells expressing MyoD. The sections were washed in 0.1 M PBS, and mounted in Vectashield mounting medium with DAPI (Vector Lab, CA) to visualize the nuclei. Immunofluorescence-stained sections were viewed on a conventional fluorescent microscope (BX60, Olympus, Tokyo, Japan) using the 20X objective. Images were captured on a digital device CCD camera system (DP-70, Olympus, Tokyo, Japan). We calculated the number of cells in each category (myoD-positive/pax7-positive, myoD-positive/pax7-negative, myoD-negative/pax7-positive). For each rat, three cross sections were counted and categorized by immunostaining.

### Statistics

All values are expressed as means ± standard deviations. Dunnett's multiple comparison test was performed to compare the torque and protein levels prior to, and on days 2, 5, and 7 after the LC. One-way analysis of variance (ANOVA) followed by Bonferroni test was used to test for differences in the body mass and muscle wet mass between the groups. Chi square test was used for analysis of the immunoreactive positive cells. Significance level was set at *P* < 0.05.

## Results

### Body weight of rats and the wet weight of triceps surae muscles

As shown in Table 1, no significant changes were observed in either, the body weight or the wet weights of the medial gastrocnemius, lateral gastrocnemius, plantaris, and soleus muscles at the different time points.

**Table 1:** Body weight and muscle wet weight

	**Pre (n = 6)**	**Day 2 (n = 6)**	**Day 5 (n = 6)**	**Day7 (n = 6)**
Body mass (g)	262.9 ± 19.6	262.2 ± 11.0	270.6 ± 12.4	260.1 ± 17.3
Medial gastrocnemius (mg)	681.0 ± 23.5	687.4 ± 47.4	667.7 ± 73.9	645.4 ± 71.9
Lateral gastrocnemius (mg)	608.8 ± 46.8	640.4 ± 60.7	623.6 ± 27.2	636.8 ± 58.4
Soleus (mg)	97.1 ± 15.4	98.0 ± 11.0	106.9 ± 5.3	103.4 ± 16.5
Plantaris (mg)	267.0 ± 21.9	269.3 ± 16.4	284.5 ± 13.1	279.0 ± 46.0

Values are means and SDs.

### Isometric tetanic torque of the ankle joint after the lengthening contraction

**Figure 1:**
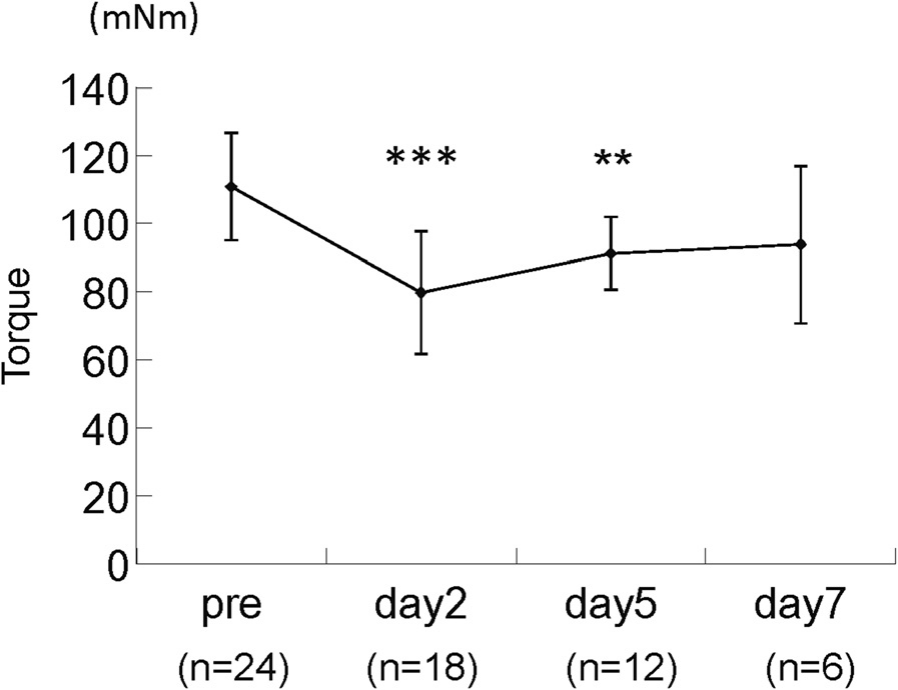
**Time course of changes in isometric torque after a lengthening contraction (LC).** Isometric tetanic torque was examined before and after LC-induced muscle injury. **: p < 0.01, ***: p < 0.001, compared to pre-treatment values. Values are means and SDs.

Time course change of isometric tetanic torques during LC is shown in Figure 1. The torque gradually decreased and showed significantly lower values on days 2 (*p* < 0.001) and 5 (*p* < 0.01) as compared to the pre-treatment control value, and on day 7.

### Protein levels of muscles

**Figure 2:**
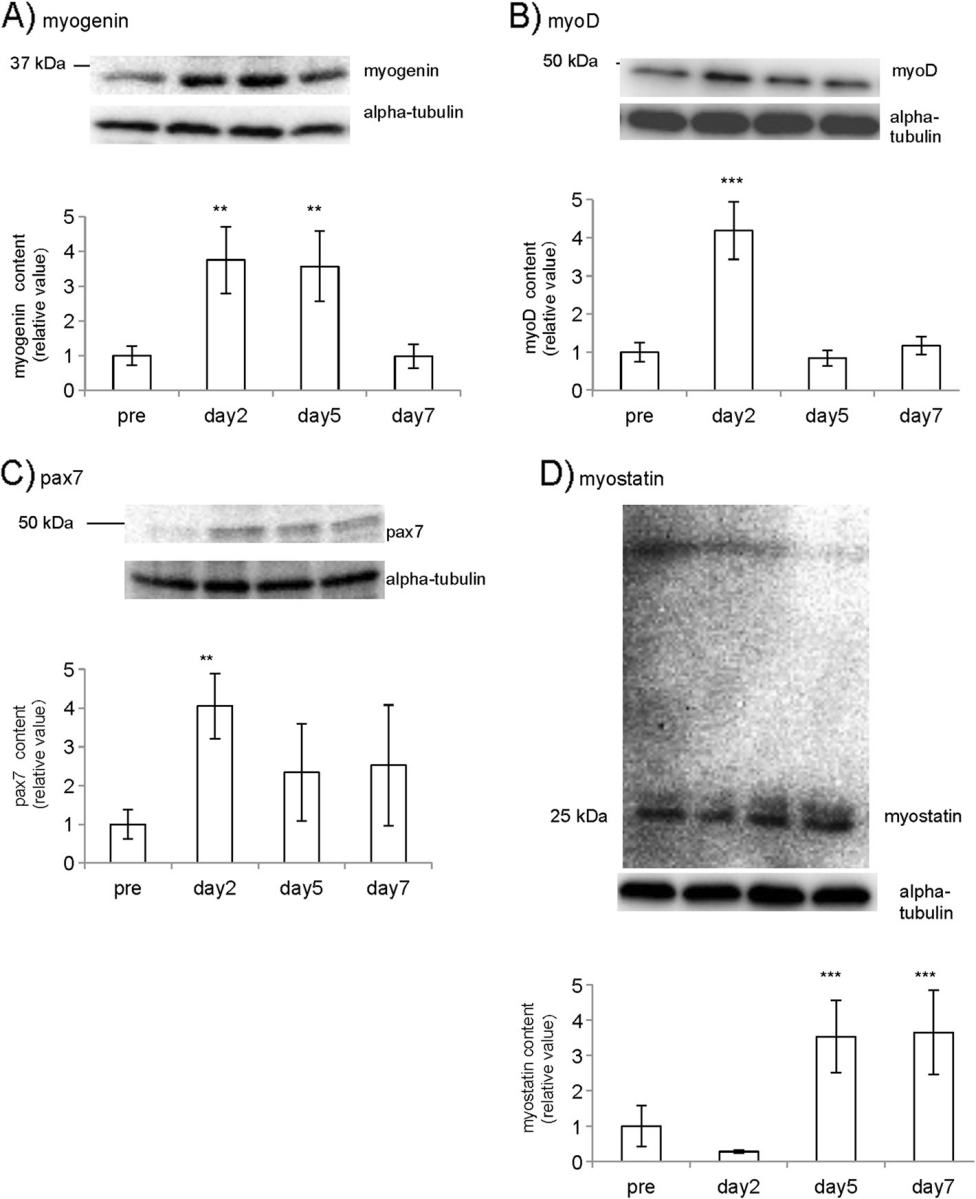
**Muscle contents of myogenin, myoD, pax7, and myostatin as determined by Western blotting.** The expression of myogenin before and after LC-induced muscle injury was examined. Obtained bands and their quantifications are shown (**A**). The expression of myoD (**B**), pax7 (**C**), myostatin (**D**) were also examined. Details are described in the materials and methods section. **: p < 0.01, ***: p < 0.001, compared to pre-treatment levels. Values are means and SDs.

We measured the muscle contents of myogenin, myoD, pax7, and myostatin to assess the process of muscle regeneration. The expression of myogenin on days 2 and 5 showed significant increases compared with that of pre-treatment value (days 2 and 5, *p* < 0.01; Figure 2A). The tissue content of myoD on day 2 was significantly higher than that of pre-treatment value (days 2, *p* < 0.001; Figure 2B). Similarly, the tissue content of pax7 also showed an increase on day 2 (*p* < 0.01; Figure 2C). However, myostatin levels were not significantly different on day 2, while those on days 5 and 7 were significantly higher than that in the pre-treatment value (days 5 and 7, *p* < 0.001; Figure 2D). With regard to myostatin, we observed multiple bands in the blot, as reported by others [28,29]. Since we confirmed that the signal intensities of 25kD were highest, we showed them as representative data.

### Localization of pax7, myoD, and myogenin

**Figure 3:**
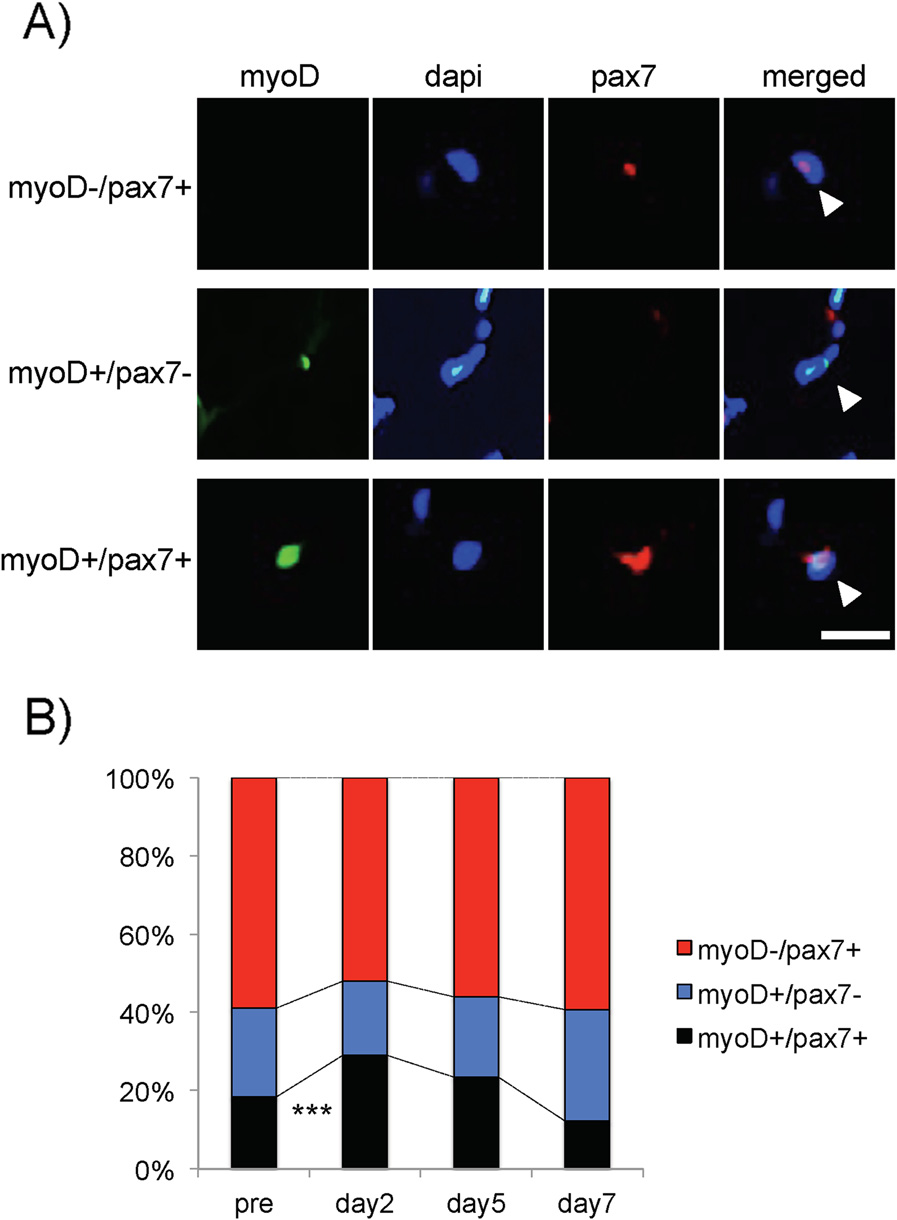
**Typical photomicrographs showing the localizations of myoD and pax7 at day 2 and their quantifications.** Triple immunostaining (**A**) of myoD (green), laminin (red), nuclei (blue), and merge for myoD-negative/pax7-positive, myoD-positive/pax7-negative, and myoD-positive/pax7-positive. Arrows indicate immunoreactive positive regions. Bar = 30 μm. The graph (**B**) shows the percentages of myoD-negative/pax7-positive cells (red bar), myoD-positive/pax7-negative (blue bar), and myoD-positive/pax7-positive (black bar). ***: p < 0.001, compared to pre-treatment and day 7 levels of the percentages of myoD-positive/pax7-positive cells.

We performed immunohistochemistry of myoD and pax7 on pre, days 2, 5, and 7 after LC-induced injury (Figure 3A). The number of myoD-positive/pax7-positive cells on day 2 was significantly higher than that of pre and day 7 (*p* < 0.001; Figure 3B). On the other hand, that on day 5 and 7 did not change compared to pre LC value. The number of myoD-positive/pax7-negative cells on day 7 inclined to increase.

## Discussion

**Figure 4:**
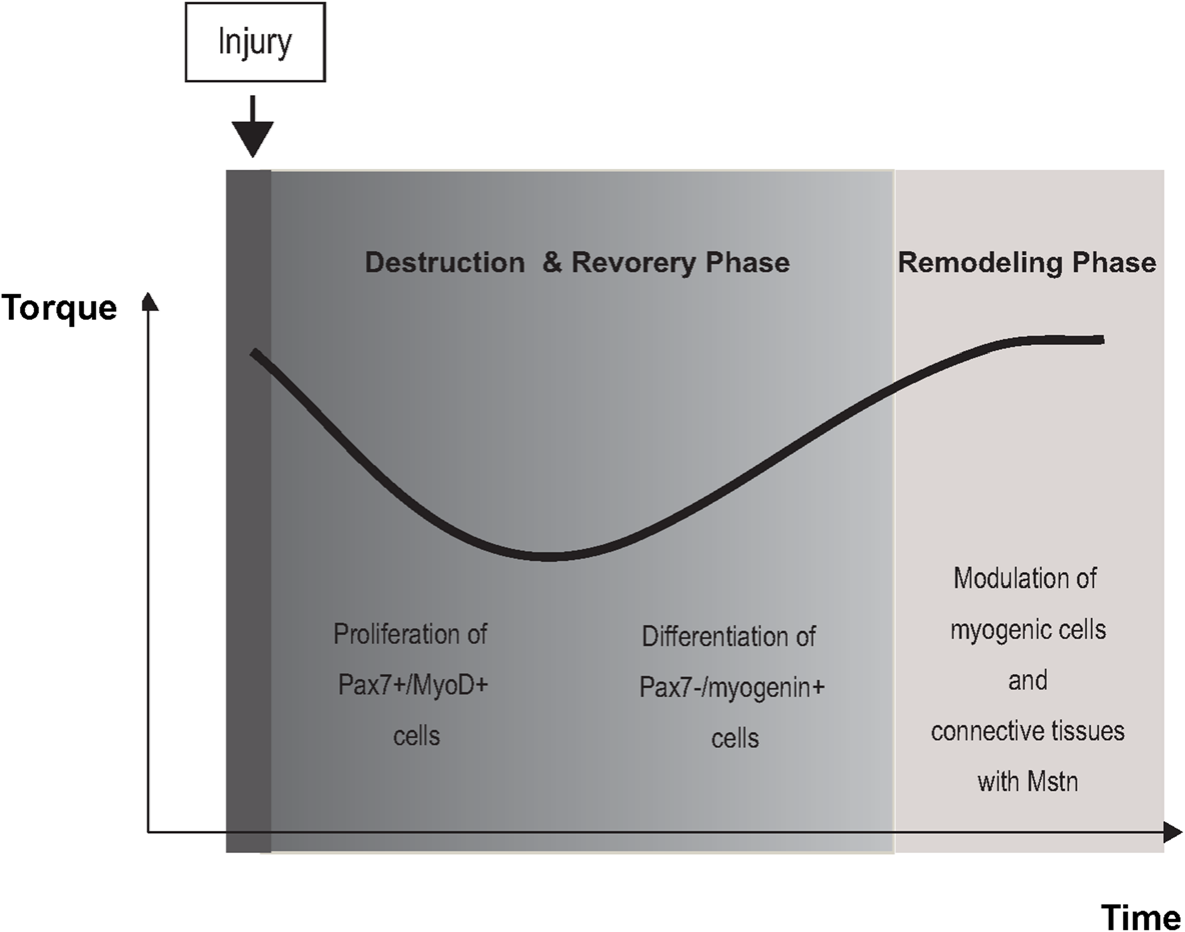
**Schematic of the 3 recovery phases following a LC-induced muscle injury.** Based on our results on the isometric torque change, we approximated the regeneration process into 3 phases including destruction, recovery (days 0–5), and remodeling (days 5–7).

The purpose of this study was to investigate the relationship between states of myogenic cells and functional recovery after LC-induced muscle injury. We measured the isometric tetanic torque of the ankle joint to evaluate muscle function. Temporal and spatial expression patterns of pax7, MRFs (myoD and myogenin), and myostatin were also examined using immunoblotting. In this discussion section, we will discuss the relationship between the functional changes and the alterations in myogenic cells during the 3 phases of muscle regeneration, i.e., destruction, regeneration, and remodeling [9]. Our working hypothesis for the time course of recovery following a LC-injury is proposed in Figure 4.

We confirmed that the isometric tetanic torque significantly decreased until day 5 and recovered to almost the control level by day 7. On day 2 after LC, it decreased by 28.8% compared to the pre-treatment value. These results are almost consistent with Song et al., who employed similar equipment for LC-induced injury [8]. Based on the time course change in muscle function, the destruction phase, the regeneration phase, and the remodeling phase are days 0–2, days 2–5, and days 5–7 after LC, respectively. In comparison with other injury models such as the myotoxic and contusion injury models, muscle strength decreased for 2–7 days after the injury, and recovered after > 14 days [26,30]. We have also reported a >7 days recovery time when larger numbers of LCs were induced [27]. Although the damage induced in this study was not as severe as compared to that in the aforementioned studies and actual muscle strain injuries, we can delineate the recovery process after muscle injury on the basis of changes in the isometric torque.

On day 2 after the LC-induced injury, the contents of myoD, myogenin, and pax7 were significantly increased. An increase in the expression of myoD and myogenin during skeletal muscle regeneration has been well documented in contusion and myotoxin treatment-induced muscle injury models [31-33]. Furthermore, we also examined pax7 expression to study the characteristics of myoD-positive nuclei (Figure 3). Immunohistochemical staining revealed the presence of 3 cell populations on day 2 after the LC; myoD-positive/pax7-positive, myoD-positive/pax7-negative, and myoD-negative/pax7-positive cells, consistent with a previous report on a cultured myotube model by Zamitt et al. [16]. Since myoD-positive/pax7-positive cells are regarded as activated satellite cells [16,17], we believe that most of these cells would subsequently differentiate to regenerate muscle fibers during the destruction and early recovery phases.

On days 5 and 7 after the LC-induced injury, immunoblotting analysis revealed that contents of myogenin and myostatin were significantly higher than the pre-treatment values. Pax7 and myogenin expression in individual cells have been reported to occur in a mutually exclusive pattern [16,34], as we confirmed in Figure 2. Since Olgun et al. showed that myogenin is critical for pax7 downregulation in differentiating cells [15], we hypothesize that the myogenin-positive cells are further committed myoblasts. Since the content of myoD on day 5 was not significantly higher than the pre-treatment value in immunoblotting analysis, this suggests that committed myoblasts mainly contribute to form nascent muscle fibers in the late recovery phase.

Immunoblotting showed that the content of myostatin was higher on days 5 and 7, and this defined the late recovery and remodeling phases. Although the number of contractions was significantly different, we previously found that myostatin contents elevated higher on day 7 after 20 LCs [27]. Myostatin is a member of the TGF-β superfamily and is known to maintain SC quiescence and to reduce myoblast recruitment and differentiation [21,35]. Previous studies suggested that myostatin in regenerating muscle fibers played a role in modulating phagocytosis and inflammation [36]. On the other hand, we observed that some myostatin positive cells also expressed pax7 (personal observation), suggesting that myostatin might also play a role in down-regulating myogenic regulatory factors to lead commitment cells to a quiescent, undifferentiated phenotype. Although authors have not explicitly commented, several reports showed that immunoreactive signals of myostatin antibody localized tissues other than myofibers [36,37]. Since the loss of myostatin and/or the inhibition of smad-mediated signaling cause extensive muscle hypertrophy [23,38], we would like to raise the possibility that myostatin negatively regulate myogenic cells to prevent hypercellularity in the late recovery and remodeling phases.

TGF-β and its superfamily are also known to induce connective tissue formation [18-20,22]. We suggest that the higher expression of myostatin on days 5 and 7 after LC-induced injury results in the production of collagen and other extracellular matrices for the remodeling and repair of connective tissue. Yong et al. found that cardiotoxin-injured mouse muscle fibers expressed TGF-β1 within 5 days after the injury. After 5 days, TGF-β expressing myofibers were replaced by fibrotic mononuclear cells [19]. Especially, fibrotic mononuclear cells might be a fibroblast cells and mainly produce collagen and other extracellular matrices. We hypothesize that myostatin mainly inhibit the proliferation of myogenic cells. The expression of TGF-β1 needs to be examined further, in order to understand the formation of connective tissue in the remodeling phase. We also consider that extracellular matrix proteins, such as tenascin-C and decorin, might regulate satellite cell proliferation and tissue regeneration [39-41].

Cares must be taken in the comparison between immunoblot and histochemical data because the thin sections were obtained from mid-belly and the specimens for SDS-PAGE were extracted from the whole muscle. Further, the isometric torque reflects the whole muscle characteristics. Since the skeletal muscle is heterogeneous tissue at the point of both metabolic and structural properties [42], comparisons of these parameters should be performed using samples collected from similar and appropriate parts of the muscle. For example, since our previous study confirmed that lengthening contraction damages were frequently seen near the musculotendinous junction, histochemical analysis for proximal and distal region may be performed to evaluate muscle damage and recovery [8]. On the other hand, we found that immunochemical observation and pathological findings were almost temporally associated. Further, we also found that a change in the isometric torque showed temporal association with these molecular expressions. Taken together, we consider that the mechanical characteristics of the muscle show some association with the molecular events. These associations also suggest that the unfamiliar eccentric contractions in this study may exert microtraumas in the whole region of the gastrocnemius, as usually occurs during regular exercise.

## Conclusions

We examined the relationship between the expression of myogenic proteins and functional changes in the skeletal muscle after a single bout of LC *in vivo*. Based on the isometric torque change, we roughly divided the regeneration process into 3 phases including destruction, recovery (days 0–5), and remodeling (days 5–7). On day 2 after the injury, significantly higher expressions of pax7 and myoD were observed. Our results also revealed myoD-positive/pax7-positive cells on day 2, suggesting that activated satellite cells play a role in the destruction and the early recovery phases. On day 5, myogenin level was significantly higher, suggesting that committed myoblasts mainly contribute in the late recovery phase. On days 5 and 7, a significantly high expression of myostatin was observed. We consider that myostatin inhibit to become hypercellularity in the remodeling phase. In conclusion, myogenic events are associated with torque recovery after LC-induced injury.

## Competing interests

The authors declare that they have no competing interests.

## Authors’ contributions

KN, EO, HS, and KH designed the study. EO and HS carried out experiment about muscle function. EO, HS and KL carried out the protein expression and localization studies. KN, EO and HS drafted the manuscript. All authors read and approved the final manuscript.

## Pre-publication history

The pre-publication history for this paper can be accessed here:

http://www.biomedcentral.com/1471-2474/13/211/prepub
